# Has aidi injection the attenuation and synergistic efficacy to gemcitabine and cisplatin in non-small cell lung cancer? A meta-analysis of 36 randomized controlled trials

**DOI:** 10.18632/oncotarget.13617

**Published:** 2016-11-25

**Authors:** Zheng Xiao, Chengqiong Wang, Ling Chen, Xuemei Tang, Lianhong Li, Nana Li, Jing Li, Qihai Gong, Fushan Tang, Jihong Feng, Xiaofei Li

**Affiliations:** ^1^ Evidence-Based Medicine Center, MOE Virtual Research Center of Evidence-based Medicine at Zunyi Medical College, Affiliated Hospital of Zunyi Medical College, Zunyi 563003, Guizhou, China; ^2^ Department of Respiratory Medicine (Center for Evidence-Based and Translational Medicine of Major Infectious Diseases), Affiliated Hospital of Zunyi Medical College, Zunyi 563003, Guizhou, China; ^3^ School of pharmacy, Zunyi Medical College, Zunyi 563003, Guizhou, China; ^4^ Department of Oncology, Affiliated Hospital of Zunyi Medical College, Zunyi 563000, Guizhou, China; ^5^ Department of Neurology, First People's Hospital of Zunyi City and Third Affiliated Hospital of Zunyi Medical College, Zunyi 563002, Guizhou, China; ^6^ Department of Parasites, Zunyi Medical College, Zunyi 563003, Guizhou, China; ^7^ Grade 2012 students, Department of Public Health, Zunyi Medical College. Zunyi 563002, Guizhou, China

**Keywords:** aidi injection, non-small cell lung cancer (NSCLC), gemcitabine and cisplatin (GP), randomized controlled trial, meta-analysis

## Abstract

Gemcitabine and cisplatin is the first line chemotherapy for non-small cell lung cancer with high toxicity. Aidi injection is a cantharidin and astragalu-based Chinese herbs injection in China. Has Aidi injection attenuation and synergistic efficacy to GP in NSCLC? There is lack of strong evidence to prove it. To further reveal it, we systematically evaluated all related studies. We collected all studies about Aidi injection plus GP for NSCLC in Medline, Embase, Web of Science, CNKI, VIP, Wanfang Database, CBM, CCRCT, Chi-CTR, and US-clinical trials (established to June 2015). We evaluated their quality according to the Cochrane evaluation handbook of randomized controlled trials (5.1.0), extracted data following the PICO principles and synthesized the data by Meta analysis. Thirty six RCTs with 2582 NSCLC patients were included, with general methodological quality in most trials. The RR values and their 95% CI of Meta-analysis for ORR, DCR and QOL were as following: 1.28 (1.17, 1.39), 1.11(1.07, 1.15) and 1.81 (1.61, 2.03). The merged RD values and their 95% CI of Meta-analysis for myelosuppression, neutropenia, thrombocytopenia, neurotoxicity and nausea and vomiting were as *following*: -0.23(-0.29, -0.17), -0.17(-0.22, -0.11), -0.13(-0.18, -0.08), -0.06(-0.17, 0.05) and -0.15(-0.21, -0.10). To compare with GP alone, *all differences were statistically significant.* The available evidence indicates that Aidi injection plus GP can significantly increase the clinical efficacy and improve the QOL of patients with NSCLC. Aidi injection can reduce myelosuppression, neutropenia, thrombocytopenia neurotoxicity and nausea/vomiting. These indirectly reveal that Aidi injection has the attenuation and synergistic efficacy to GP chemotherapy in NSCLC.

## INTRODUCTION

Lung cancer continues to be a major global health problem, which is diagnosed in more than 1.6 million new patients each year [1]. Approximately 80% of lung cancers are non-small cell lung cancer (NSCLC) *and* over 50% of patients with NSCLC *have* advanced local invasion and distant metastasis [2]. *Hence, they* are forced to accept chemotherapy, radiotherapy or immunotherapy because losing the chance of surgery [3–4]. Gemcitabine and cisplatin (GP) is the first line chemotherapy and it can increase the overall response rate *(ORR)* of NSCLC patients [5–7]. But its clinical efficacy is often limited by the acute or subacute toxicity including leucopenia, anemia and thrombocytopenia, et.al [8–9]. *All these lead to poor clinical efficacy and substandard quality of life for patients (QOL). Therefore, finding ways to alleviate the toxicity and improve the clinical efficacy and quality of life for patients becomes a key issue.*

Aidi injection (Z52020236, China Food and Drug Administration) is a cantharidin and astragalu-based Chinese herbs injection in China. It is composed by the extracts of Cantharidin, Astragalus, senticosus Eleutherococcus and Ginseng. They are important anticancer Chinese medicinal herbs which appear to have anti-tumor activity, reduce the acute or subacute toxicity and up-regulate the tumor immunity [10–13]. Can Aidi injection alleviate the toxicity induced by GP chemotherapy in NSCLC? Has it attenuation and synergistic efficacy to GP in NSCLC? Related studies [14–16] had shown that Aidi injection plus GP might improve the clinical efficacy and Aidi injection might alleviate the toxicity in NSCLC. Unfortunately, these conclusions were different in different studies with lower sample size. *There* is lack of strong evidence to prove it. *To further reveal whether Aidi injection has attenuation and synergistic efficacy to GP in NSCLC, we systematically evaluated all related studies.*

## RESULTS

### Search results

**Figure 1:**
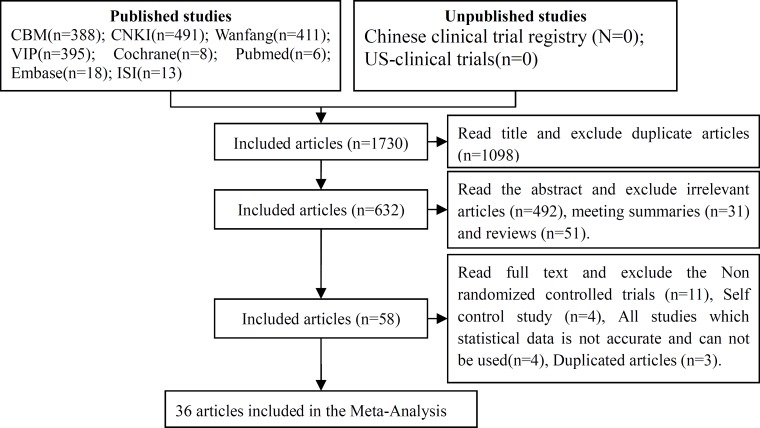
Articles retrieved and assessed for eligibility

The initial database search identified 1730 *published studies* using our search strategies, *and* unpublished studies were not retrieved (Figure 1). After successively applying the study exclusion criteria, 36 RCTs were included.

### Characteristics of the included studies

Thirty six RCTs with 2582 NSCLC patients *in* China were included in this meta-analysis (Table 1). The cases of Aidi injection plus GP and GP were 1319 and 1263 respectively. The male and female were 1606 and 906 respectively with age range between 21 and 86 years. The dosage of Aidi injection was 50-100ml/day and treatment time was 2-3 weeks and 2-3 cycles by intravenous injection. *Clinical efficacy* was evaluated by ORR, DCR and QOL. *Drug toxicity* was evaluated by hematotoxicity, liver injury, renal injury, neurotoxicity and nausea/vomiting.

**Table 1:** Characteristics of the included studies

First author. Year	Patients(III-IV)			Interventions		Outcomes		
	E/C	M/F	Age	AD &GP (D/T/C)	C	O1	O2	O3
Zou, Y.et al2006 [17]	42/39	56/25	35-73	80ml/14d/2-4	GP	√	√	√
Feng, X, et.al 2008 [18]	68/62	88/42	38-74	50ml/15d/2	GP	√	√	√
Sun, G.et.al2008 [19]	33/30	54/9	34-73	100ml/14d/2	GP	√	-	√
Yang, Q, et.al 2008 [20]	30/27	39/18	34-82	80ml/8d/2	GP	√	√	-
Zhao, H.et.al2008 [21]	30/20	31/19	29-73	30ml/21d/3	GP	√	√	-
Lv, D, et.al2009 [22]	30/30	42/18	45-70	80ml/10d/2	GP	√	√	√
Song, Z, et.al2009 [23]	30/30	36/24	53-76	50ml/14d/2	GP	√	√	√
Wang, Y2009 [24]	32/27	48/11	-	−1-10d/2	GP	√	-	√
Wen, K, et.al 2009 [25]	38/38	52/24	32-77	50ml/8-10d/2	GP	√	√	√
Zhang, L2009 [14]	32/31	44/19	31-79	80ml/14d/2	GP	√	√	√
Hong, Y, et.al 2010 [26]	90/70	82/78	38-70	60ml/14d/2	GP	√	√	√
Hou, A, et.al 2010 [27]	40/38	49/29	32-79	50ml, 14d,2	GP	√	√	√
Li, Z, et.al 2010 [28]	36/36	39/33	29-75	50-100ml/15d/2	GP	√	√	√
Liu, Y, et.al 2010 [29]	32/32	37/27	45-75	50ml/14d/4	GP	√	-	√
Shi, X, et.al 2010 [30]	28/28	47/9	48-72	50ml/14d/2	GP	√	√	√
Ding, P, et.al 2011 [15]	18/22	27/13	-	50ml/10d/2	GP	√	-	-
Fan, S, et.al 2011 [31]	41/38	54/25	39-73	50ml/21d/2-4	GP	√	√	-
He, W, et.al 2011 [32]	29/23	29/23	21-74	50-100ml/15d/2-3	GP	√	√	√
Jiang, S, et.al 2011 [33]	32/30	39/23	60-75	100ml/14/2	GP	√	-	-
Lu, Z, et.al 2011v [34]	34/34	39/29	40-76	100ml/14d/2	GP	√	-	√
Wu, Q, et.a'2011 [34]	30/30	41/19	45-77	100ml/16d/2	GP	√	-	-
Fu, L, et.al2012 [36]	35/35	-	61-84	50ml/14d/2	GP	√	-	-
Pei, W2012 [37]	40/40	47/33	39-72	50ml/8d/2	GP	√	-	-
Sun, J, et.al2012 [38]	34/34	42/26	60-86	50ml/10d/2	GP	√	√	√
Wang, J, et.al2012 [39]	25/24	35/14	-	60ml/14d/2	GP	√	-	√
Wang, Y, et.al2012 [40]	36/36	46/26	32-74	80ml/10d/2	GP	√	√	√
Xu, Y, et.al2012 [41]	33/33	36/30	-	80mg/10d/4	GP	√	-	√
Zhang, Y2012 [42]	41/42	63/20	-	60ml/14d/3	GP	√	-	√
Cai, H, et.al2013 [43]	19/19	21/17	36-68	50-100ml/15d/2	GP	-	-	√
Ju, S, et.al2013 [44]	34/34	36/32	61-81	50ml/14d/2	GP	√	√	√
Lai, L2013 [45]	70/70	73/67	45-79	50ml/14d/2	GP	√	√	√
Xu, H, et.al2013 [46]	38/42	55/25	39-81	50ml/14d/3	GP	√	√	√
Li, J, et.al2014 [47]	27/27	32/22	34-68	50ml/8-10d/4	GP	√	-	√
Liu, Y, et.al2014 [16]	43/43	53/33	39-73	50ml/8-10d/2	GP	√	-	√
Liu, Z, et.al2014 [48]	24/24	30/18	35-80	60ml/21d/2	GP	-	√	-
Wen, H2014 [49]	45/45	64/26	61-81	50ml/21d/2	GP	√	√	√

Note: Patients: Non-small cell lung cancer (NSCLC); E: Experimental group(Aidi injection plus GP); C: Control group(GP); M: male, F: female; AD: Aidi injection; D/T/C: Dose/times/Cycles; Outcome: O1:Clinical efficacy included ORR and DCR; O2: quality of life (QOL); O3: Acute/subacute toxicity included hematotoxicity, liver injury, renal injury, nausea and vomiting and neurotoxicity.

### Methodological bias of the included studies

**Figure 2:**
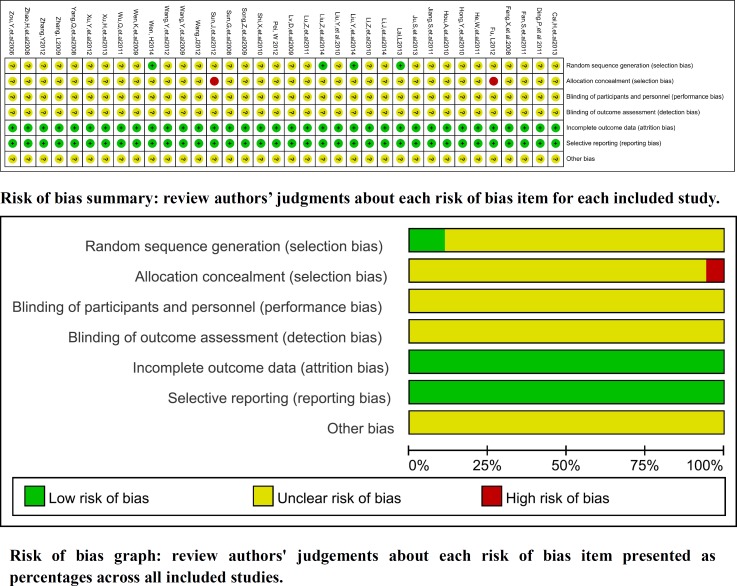
Risk of methodological bias of the included studies

In 36 trials, the methods of random allocation were described clearly in only 4 trials. This indicated that there was selectivity bias in the included-studies. *The random allocation concealment was open by hospitalization orders respectively in two trials. Not all the included studies were described as blinding to patients and doctor. Therefore, it indicated that there were selective bias* and implementation bias. All data were complete *and selective report did not appear in all of the studies.* Other bias was not clear. Characteristics and quality of all included studies are presented in Figure 2.

### Clinical efficacy

**Figure 3:**
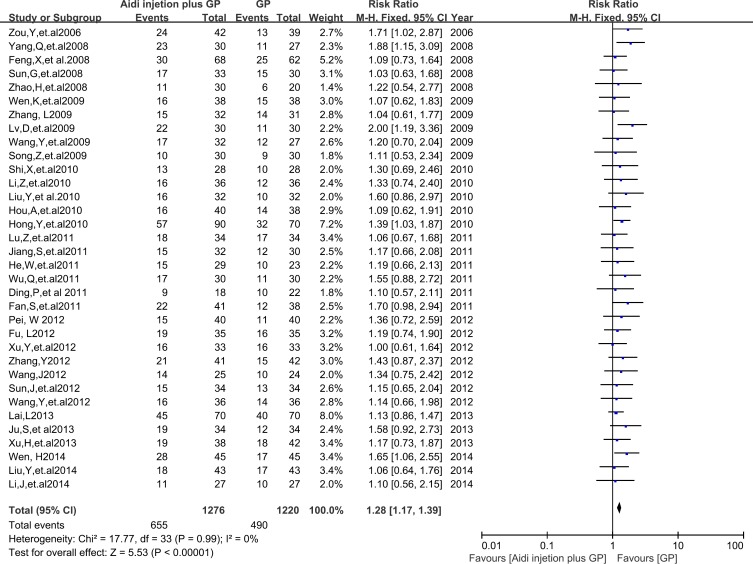
The analysis of ORR between two groups

In 36 RCTs, 34 trials with 2496 cases were included (Figure 3). There was homogeneity between studies according to the heterogeneity test (Chi^2^=17.77, P=0.99, I^2^=0%). Meta-analysis showed that the ORR was statistically different between the two groups [1.28 (1.17, 1.39), P < 0.00001)] by fixed-effects model.

**Figure 4:**
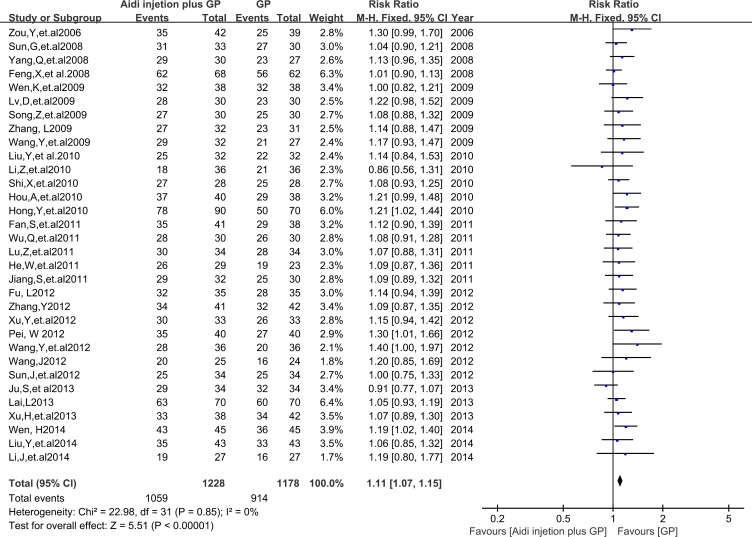
The analysis of DCR between two groups

Thirty-two trials with 2406 cases were included (Figure 4). There was homogeneity between studies (Chi^2^=22.98, P = 0.85, I^2^ = 0%). Meta-analysis showed that the DCR was statistically different between the two groups [1.11(1.07, 1.15), P<0.00001) by fixed-effects model. *To compare with chemotherapy alone, all results showed that* Aidi injection plus GP could significantly improve the ORR and DCR of patients with NSCLC.

### QOL evaluation

**Figure 5:**
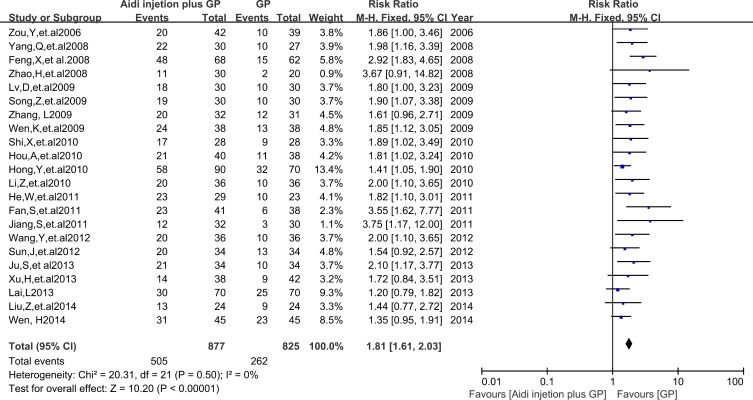
The analysis of QOL between two groups

In 36 RCTs, 22 trials with 1702 cases were included (Figure 5). There was homogeneity between studies (Chi^2^=20.31, P=0.50, I^2^=0%). Meta-analysis showed that the QOL was statistically different between the two groups [1.81 (1.61, 2.03), P<0.00001] by fixed-effects model. To compare with chemotherapy alone, result showed that Aidi injection plus GP could significantly improve the QOL of patients with NSCLC.

### Acute/subacute toxicity

### Hematotoxicity

**Figure 6:**
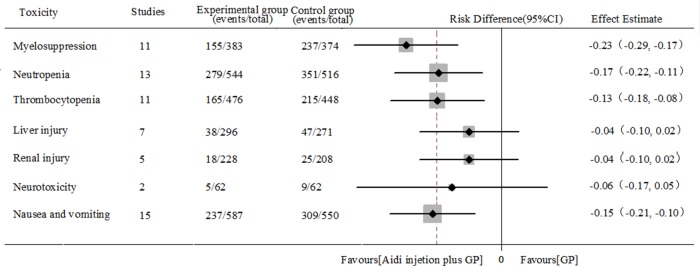
Meta analysis results of toxicity between two groups

In 36 RCTs, 11 trials with 757 cases were included (Table 2, Figure 6 and Supplementary Figure S1). There was homogeneity between studies (Chi^2^=9.35, P=0.50, I^2^ = 0%). Meta-analysis showed that the risk difference(RD) of myelosuppression was statistically different between the two groups [−0.23(-0.29, -0.17), P<0.05] by fixed-effects model.

**Table 2:** Meta analysis results of toxicity between two groups

Toxicity	Study	E	C	I2	Effect Estimate RD(95%CI)	P	SM	PB
**Myelosuppression**	11	155/383	237/374	0%	−0.23(-0.29, -0.17)	<0.05	FEM	no
**Neutropenia**	13	279/544	351/516	49%	−0.17(-0.22, -0.11)	<0.05	FEM	yes
**Thrombocytopenia**	11	165/476	215/448	17%	−0.13(-0.18, -0.08)	<0.05	FEM	yes
**Liver injury**	7	38/296	47/271	0%	−0.04(-0.10, 0.02)	=0.16	FEM	unclear
**Renal injury**	5	18/228	25/208	0%	−0.04(-0.10, 0.02)	=0.18	FEM	unclear
**Neurotoxicity**	2	5/62	9/62	21%	−0.06(-0.17, 0.05)	=0.25	FEM	unclear
**Nausea/vomiting**	15	237/587	309/550	1%	−0.15(-0.21, -0.10)	<0.05	FEM	no

Note: E: Experimental group (events/total); C: Control group (events/total); RD: Risk Difference; CI: Confidence interval; SM: Statistical method; FEM: Fixed-effects model; PB: Publication bias; Forest of all results are in Appendix(Supplementary Figures S1–S7).

In 36 RCTs, 13 trials with 1060 cases were included (Table 2, Figure 6 and Supplementary Figure S2). There was homogeneity between studies (Chi^2^=23.41, P=0.02, I^2^=49%). Meta-analysis showed that the neutropenia was statistically different between the two groups [−0.17(-0.22, -0.11), P<0.05] by fixed-effects model.

In 36 RCTs, 11 trials with 924 cases were included (Table 2, Figure 6 and Supplementary Figure S3). There was homogeneity between studies (Chi^2^=12.05, P=0.28), I^2^=17%). Meta-analysis showed that the thrombocytopenia was statistically different between the two groups [−0.13(-0.18, -0.08), P<0.05] by fixed-effects model. To compare with chemotherapy alone, results showed that Aidi injection plus GP could significantly reduce myelosuppression, neutropenia and thrombocytopenia of patients with NSCLC.

### Liver and renal injury

In 36 RCTs, 7 trials with 567 cases were included (Table 2, Figure 6 and Supplementary Figure S4). There was homogeneity between studies (Chi^2^=2.21, P = 0.90, I^2^ = 0%). Meta-analysis showed that the liver injury was no statistically different between the two groups [−0.04(-0.10, 0.02), P=0.16] by fixed-effects model.

In 36 RCTs, 5 trials with 436 cases were included (Table 2, Figure 6 and Supplementary Figure S5). There was homogeneity between studies (Chi^2^ = 0.38, P = 0.98, I^2^ = 0%). Meta-analysis showed that the renal injury was no statistically different between the two groups [−0.04(-0.10, 0.02), P=0.18] by fixed-effects model. *To compare with chemotherapy alone, none of the results supported* that Aidi injection plus GP could reduce the liver and renal injury of patients with NSCLC.

### Other toxicity

In 36 RCTs, 2 trials with 124 cases were included (Table 2, Figure 6 and Supplementary Figure S2). There was homogeneity between studies (Chi^2^=1.26, P=0.26, I^2^=21%). Meta-analysis showed that the neurotoxicity was no statistically different between the two groups [−0.06(-0.17, 0.05), P=0.25] by fixed-effects model.

In 36 RCTs, 15 trials with 1137 cases were included (Table 2, Figure 6 and Supplementary Figure S7). There was homogeneity between studies (Chi^2^=14.21, P=0.43, I^2^=1%). Meta-analysis showed that the nausea and vomitting was statistically different between the two groups [−0.15(-0.21, -0.10), p<0.05] by fixed-effects model. To compare with chemotherapy alone, all results showed that Aidi injection plus GP could significantly reduce the nausea and vomiting of patients with NSCLC, not the neurotoxicity.

### Publication bias and sensitivity analysis

### Publication bias analysis

**Figure 7:**
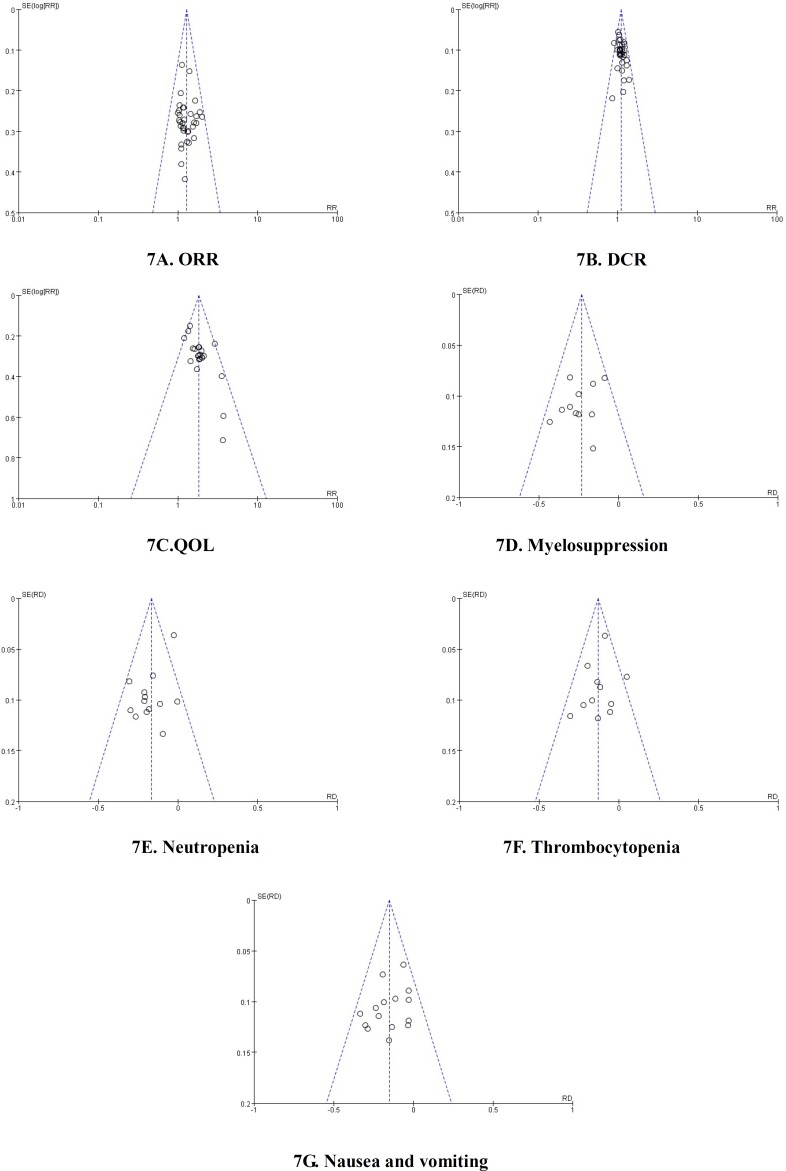
Publication bias analysis

The funnel plots were symmetric in the studies about ORR, QOL, myelosuppression and nausea and vomiting (Figure 7A, 7C, 7D and 7G). This indicated that there was no publication bias in these studies which objectively reported the results. The funnel plots were significantly asymmetric in the studies about the DCR, neutropenia, thrombocytopenia (Figure 7B, 7E and 7F). *Results showed that all points were asymmetric and some points were distributed outside of the funnel. This* indicated that there was publication bias *in the included-studies*. The DCR [44] was underestimated and the neutropenia [18] and thrombocytopenia [24] were overestimated. *All of the above facts* were beneficial to the conclusion. Therefore, we didn't implement meta-analysis by excluding the *over- or underestimated* studies. In summary, *all of the results had good objectivity.*

### Sensitivity analysis

There was good homogeneity between studies in all meta-analysis. The methodological quality and sample size *had* good consistency between all included-studies. Therefore, the sensitivity was not evaluated through *deleting the high weight or poor studies.* In summary, the stability *was* good in this meta-analysis.

## DISCUSSION

In this study, 36 RCTs were included. There were 2582 NSCLC (III-IV) patients including 1606 male and 906 female patients between 21 and 86 years *of age*. The dosage of Aidi injection was 50-100ml/day and treatment time was 2-3 weeks and 2-3 cycles by intravenous injection. According to the WHO guidelines, meta-analysis showed that Aidi injection plus GP could significantly improve the ORR, DCR and QOL of patients with NSCLC. Thirty-six RCTs with 2036 NSCLC patients were included, which ensured sufficient sample size for meta-analysis. The DCR were underestimated which was beneficial to the conclusion. The methodological quality and sample size had good consistency between all included-studies. In summary, all results have good objectivity and stability. Similar meta-analysis [50]showed that Aidi injection plus paclitaxel and cisplatin could significantly improve the clinical efficacy of patients with NSCLC. Cantharidin and Astragalus, Eleutherococcus senticosus and Ginseng are important components of Aidi injection. Animal studies [51] also showed that *cantharidin plus chemotherapy had obvious inhibitive effects* on the growth of Lewis lung cancer. *In-vitro studies* [52–53]showed that cantharidin could induce the apoptosis and inhibit the proliferation, migration and invasion of A549 human lung cancer cells. Wu.S2013, et.al [54–56] reported that Aidi injection with chemotherapy could significantly improve the percentage of CD3^+^T cells, CD4^+^T cells, CD8^+^T cells and NK cells and the CD4^+^/CD8^+^ T cells ratio in peripheral blood, *and* improve tumor immunity of patient with NSCLC. Many studies [57–62] also showed that Astragalus, senticosus Eleutherococcus, and Ginseng had anti-tumor activity and immune regulation functions. So we believe that *Aidi injection plus GP can significantly increase the clinical efficacy and improve the QOL of patients with NSCLC* through inducing the cancer cell apoptosis and up-regulating tumor immunity. These studies indirectly reveal that Aidi injection has synergistic efficacy to GP in NSCLC.

### Acute/subacute toxicity

In 27 RCTs, the hematotoxicity, liver injury, renal injury, neurotoxicity and nausea/vomiting were reported according to the WHO standards [63]. Meta-analysis showed that Aidi injection could significantly reduce the incidence rate of myelosuppression, neutropenia, thrombocytopenia neurotoxicity and nausea/vomiting, *but not of the liver and renal injury.* Twenty-seven RCTs with 2036 NSCLC patients were included, which ensured sufficient sample size for meta-analysis. But there were smaller sample in the meta-analysis of liver injury, renal injury and neurotoxicity. This might lead to insufficient assessment to them. The neutropenia [18] and thrombocytopenia [24] were overestimated which were beneficial to the conclusion. The methodological quality and sample size had good consistency between all included-studies. *Overall,* all results have good objectivity and stability. Similar meta-analysis [64–65] showed that Aidi injection could decrease the toxicity induced by navelbine and cisplatin (NP) or paclitaxel and cisplatin(TP) in NSCLC. Most studies showed that Astragalus, Eleutherococcus senticosus, and Ginseng might reduce the chemotherapy related toxicity through promoting hematopoiesis [66–67] and relieving oxidative stress [68–71]. These studies provide indirect evidence for the above conclusions. In summary, Aidi injection can reduce myelosuppression, neutropenia, thrombocytopenia neurotoxicity and nausea/vomiting, but not the liver and renal injury. These indirectly reveal that *Aidi injection can attenuate the acute/subacute toxicity of GP chemotherapy in NSCLC.*

### Limitations of this study

There were some limitations in this study. Firstly, Chinese and English databases were retrieved, but not Japanese and Korean databases, all studies were published in China. *These could lead to ethnical bias.* Secondly, in 36 trials, only 4 trials described the random allocation method. The random allocation concealment and binding to patients and doctors were not described in *all of the included-studies*. *These indicated that there were selective bias and implementation bias and therefore led to the overestimation of the clinical efficacy and attenuation of the treatment group.* Thirdly, long term efficacy has not been evaluated and there were smaller sample in the meta-analysis of liver injury, renal injury and neurotoxicity. *All of these* might lead to an inadequate assessment *of* the clinical efficacy and attenuation. *All together, the quality of the included studies* is inadequate *and the results need to be* further confirmed by standardized studies including RCT or real-world studies.

## MATERIALS AND METHODS

This article followed Preferred Reporting Items for Systematic Reviews and Meta-Analyses (PRISMA) guidelines.

### Literature search strategy

Two reviewers (Xuemei Tang and Chengqiong Wang) independently searched articles in electronic databases using the search strategy (“Lung Neoplasms” [Mesh] OR Pulmonary Neoplasms OR Lung Neoplasm OR Pulmonary Neoplasm OR Lung Cancer OR Lung Cancers OR Pulmonary Cancer OR Pulmonary Cancers OR lung carcinoma OR Pulmonary carcinoma OR NSCLC) AND (aidi OR aidi injection). Published studies were retrieved in Medline, Embase, Web of Science, China National Knowledge Infrastructure Database(CNKI), Chinese Scientific Journals Full-Text Database(VIP), Wanfang Database, China Biological Medicine Database (CBM) (established to June 2015) and Cochrane Central Register of Controlled Trials (CCRCT, Issue 6 of 12, June 2015). Unpublished studies were retrieved in Chinese clinical trial registry (Chi-CTR) and US-clinical trials (established to June 2015). All retrievals were implemented by the Mesh and free word. No language restrictions were placed on the search. *Ethical approval was not required, as our study pertains only to the meta-analysis of published or unpublished studies.*

### Studies inclusion and exclusion criteria

#### Inclusion criteria

Included studies must meet the following criteria: (1) We included all studies with NSCLC (III-IV) in accordance with histopathological and cytological diagnostic criteria and non operative patients. (2)There were randomized controlled trials (RCTs) groups. (3) The experimental group was Aidi injection plus GP and *the control group* was GP.(4) *Subjects previously included in the study did not* receive other therapies including other Chinese herbs and intra-arterial infusion chemotherapy. (5) Clinical efficacy: According to the World Health Organization (WHO) guidelines [63] for solid tumor responses, indicators were complete response (CR), partial response (PR), no change (NC), progressive disease (PD), objective response rate (ORR) equals CR+PR and disease control rate (DCR) equals CR+PR+NC. *Clinical efficacy was evaluated by objective response rate (ORR) and disease control rate (DCR).* Quality of life (QOL): According to karnofsky performance score (KPS) grading system, QOL was improved *when* KPS increased 10 points after the treatment. Acute/subacute toxicity: According to WHO standards [63], the acute/subacute toxicity was evaluated by hematotoxicity including neutropenia (granulocytes<2×10^9^/L), thrombocytopenia (platelets<100×10^9^/L), and myelosuppression), liver and renal injury, neurotoxicity and nausea and vomiting. (6)Time and settings: *no restrictions were set on the duration of follow-ups or types of settings.*

### Exclusion criteria

Excluded studies must meet the following criteria: (1) Duplicated articles. (2) Unrelated studies including *other themes, and animal and in-vitro studies.* (3) Non-randomized controlled studies, (4) *Abstracts and reviews without specific data*. (5) *Studies with inaccurate information or non-usable statistical data.*

### Study quality evaluation

We evaluated the quality of all included-studies according to the Cochrane evaluation handbook of RCTs (5.1.0) [72]. *The bias parameters were the random sequence generation (selection bias), the allocation concealment (selection bias), the blinding of participants and personnel (performance bias), the blinding of outcome assessment (detection bias), the incomplete outcome data (attrition bias), the selective report (reporting bias), and the other bias.* We judged each item on three levels (“Yes” for low bias, “No” for a high risk of bias, and “Unclear”). Then, we assessed the trials and categorized them into three levels: low risk of bias (all the items were categorized “Yes”), high risk of bias (at least one item ranked “No”), and unclear risk of bias (at least one item was “Unclear”).

### Selection and evaluation of articles

Two reviewers (Nana Li and Xuemei Tang) independently selected and evaluated articles according to the above standards. Any disagreements were resolved by *discussing between themselves or with* Zheng Xiao.

### Data extraction and statistical analysis

Two reviewers (Lianhong Li and Xuemei Tang) independently extracted all data including: (1) Publishing time and country. (2) Study design including the randomization methods, demographic characteristics and blinding implementation. (3) The sample size of experimental and control group. (4) Clinical efficacy including ORR, DCR and QOL and acute or subacute toxicity including hematotoxicity, liver and renal injury, neurotoxicity and nausea and vomiting. Meta-analysis was implemented by two reviewers (Chengqiong Wang and Jing Li) with Review Manager 5.3 (The Cochrane Collaboration, Oxford, UK). The relative risk (RR), Risk difference (RD) and 95% confidence intervals (CI) were calculated. Statistical heterogeneity of the results across trials was assessed by Chi-square based Q-statistic test and the inconsistency was calculated by I^2^. If the homogeneity (P≥ 0.1, I^2^ ≤ 50%) was not rejected, the fixed-effects model was used to calculate the RR and the 95% CI. *The results were analyzed by random-effects model, if the heterogeneity (P<0.1, I^2^ > 50%) was higher and the results of the fixed and random effect model had good consistency.* The clinical heterogeneity was handled by strict inclusion and exclusion criteria and subgroups analysis. Statistical heterogeneity was reduced by random effects model if the results of the fixed and random effect model had good consistency. Otherwise, the results were analyzed by descriptive analysis. Publication bias was evaluated through funnel plots *if there were more than 10 included studies. The sensitivity was evaluated through deleting the studies with high weight, poor*, *over- or underestimated results.*

## CONCLUSIONS

To compare with GP chemotherapy, Aidi injection plus GP can significantly increase the clinical efficacy and improve the QOL *of* patients with NSCLC. Aidi injection can reduce the myelosuppression, neutropenia, thrombocytopenia neurotoxicity and nausea/vomiting, but not the liver and renal injury. These indirectly reveal that Aidi injection has attenuation and synergistic efficacy to GP chemotherapy in NSCLC. Its long-term effect is not clear. The quality of *the* included studies is inadequate. *The results need to be confirmed* by further large sample RCT or real-world studies with *longer follow-ups.*

## APPENDIX

Forest plot and Funnel plot of the toxicity between two groups(Supplementary Figures S1–S7).

## SUPPLEMENTARY FIGURES


