# Supplementary material for: Has aidi injection the attenuation and synergistic efficacy to gemcitabine and cisplatin in non-small cell lung cancer? A meta-analysis of 36 randomized controlled trials
Source: Oncotarget. 2016 Nov 25;8(1):1329–42. doi: 10.18632/oncotarget.13617 (PMC5352058; doi:10.18632/oncotarget.13617)
Supplement: Supplementary file 1 [file oncotarget-08-1329-s001.pdf]

Has aidi injection the attenuation and synergistic efficacy to gemcitabine and cisplatin in non-small cell lung cancer? A meta-analysis of 36 randomized controlled trials

SUPPLEMENTARY FIGURES

	Aidi injection plus GP		GP			Risk Difference	
Study or Subgroup	Events	Total	Events	Total	Weight	M-H, Fixed, 95% CI	Year
Zou,Y,et.al2006	30	42	34	39	10.7%	-0.16 [-0.33, 0.01]	2006
Song,Z,et.al2009	7	30	12	30	7.9%	-0.17 [-0.40, 0.06]	2009
Lv,D,et.al2009	16	30	24	30	7.9%	-0.27 [-0.50, -0.04]	2009
Li,Z,et.al2010	10	36	21	36	9.5%	-0.31 [-0.52, -0.09]	2010
Shi,X,et.al2010	16	28	23	28	7.4%	-0.25 [-0.48, -0.02]	2010
Liu,Y,et al.2010	3	32	11	32	8.5%	-0.25 [-0.44, -0.06]	2010
He,W,et.al2011	9	29	17	23	6.8%	-0.43 [-0.67, -0.18]	2011
Xu,Y,et.al2012	23	33	33	33	8.7%	-0.30 [-0.46, -0.14]	2012
Ju,S,et al2013	11	34	23	34	9.0%	-0.35 [-0.58, -0.13]	2013
Cai,H,et.al2013	5	19	8	19	5.0%	-0.16 [-0.46, 0.14]	2013
Lai,L2013	25	70	31	70	18.5%	-0.09 [-0.25, 0.08]	2013
**Total (95% CI)**		**383**		**374**	**100.0%**	**-0.23 [-0.29, -0.17]**	
Total events	155		237				
Heterogeneity: Chi² = 9.35, df = 10 (P = 0.50); I² = 0%							
Test for overall effect: Z = 7.16 (P < 0.00001)							

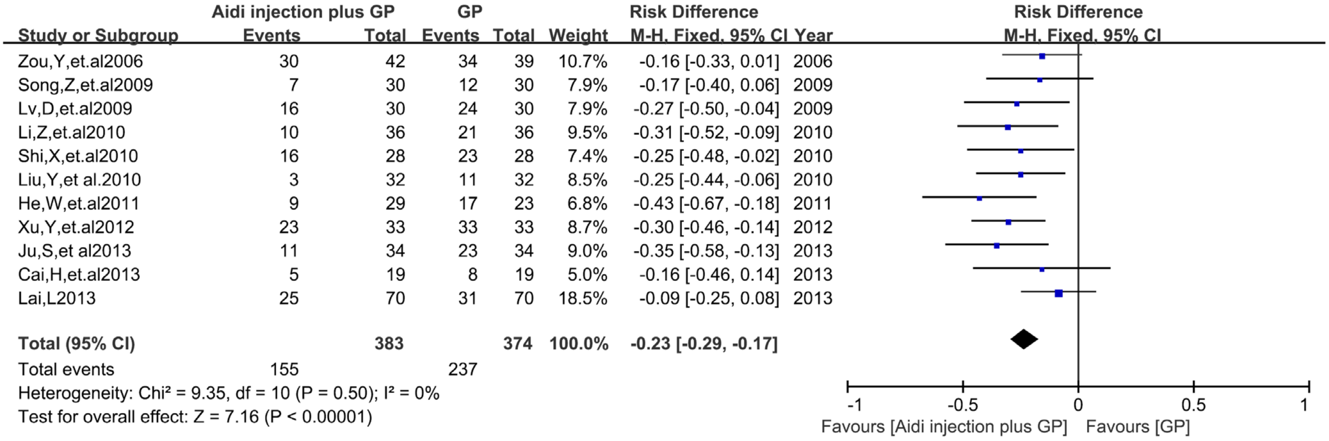

Supplementary Figure S1: The analysis of myelosuppression between two groups.

Study or Subgroup	Aidi injection plus GP Events	Aidi injection plus GP Total	GP Events	GP Total	Weight	Risk Difference M-H, Fixed, 95% CI	Year
Feng,X,et al.2008	64	68	60	62	12.3%	-0.03 [-0.10, 0.04]	2008
Wen,K,et.al2009	23	38	31	38	7.2%	-0.21 [-0.41, -0.01]	2009
Wang,Y,et.al2009	26	32	22	27	5.5%	-0.00 [-0.20, 0.20]	2009
Zhang, L2009	5	32	14	31	6.0%	-0.30 [-0.51, -0.08]	2009
Hong,Y,et.al2010	26	90	31	70	14.9%	-0.15 [-0.30, -0.00]	2010
Lu,Z,et.al2011	11	34	20	34	6.4%	-0.26 [-0.49, -0.04]	2011
Xu,Y,et.al2012	23	33	33	33	6.2%	-0.30 [-0.46, -0.14]	2012
Sun,J,et.al2012	23	34	30	34	6.4%	-0.21 [-0.40, -0.01]	2012
Zhang,Y2012	12	41	17	42	7.9%	-0.11 [-0.32, 0.09]	2012
Wang,Y,et.al2012	19	36	26	36	6.8%	-0.19 [-0.41, 0.02]	2012
Wang,J2012	7	25	9	24	4.6%	-0.09 [-0.36, 0.17]	2012
Xu,H,et.al2013	13	38	22	42	7.6%	-0.18 [-0.40, 0.03]	2013
Liu,Y,et.al2014	27	43	36	43	8.1%	-0.21 [-0.39, -0.03]	2014
**Total (95% CI)**		**544**		**516**	**100.0%**	**-0.17 [-0.22, -0.11]**	
Total events	279		351				

Heterogeneity: Chi² = 23.41, df = 12 (P = 0.02); I² = 49%
Test for overall effect: Z = 6.31 (P < 0.00001)

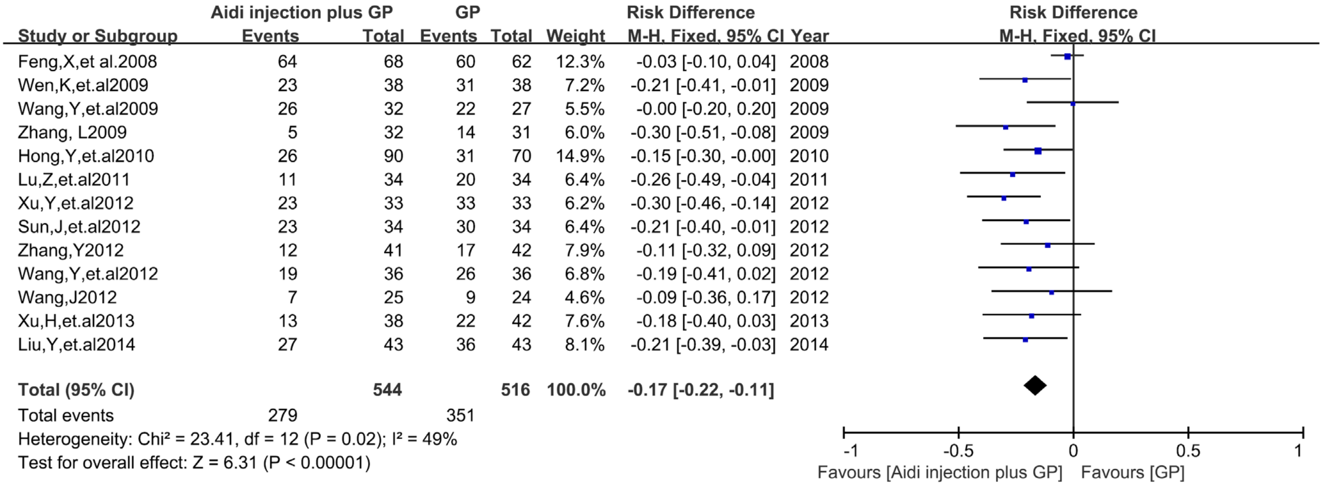

Supplementary Figure S2: The analysis of neutropenia between two groups.

Study or Subgroup	Aidi injection plus GP Events	Aidi injection plus GP Total	GP Events	GP Total	Weight	Risk Difference M-H, Fixed, 95% CI	Year
Feng,X,et al.2008	62	68	62	62	14.1%	-0.09 [-0.16, -0.02]	2008
Wang,Y,et.al2009	4	32	2	27	6.4%	0.05 [-0.10, 0.20]	2009
Wen,K,et.al2009	22	38	24	38	8.3%	-0.05 [-0.27, 0.17]	2009
Zhang, L2009	4	32	9	31	6.8%	-0.17 [-0.36, 0.03]	2009
Hong,Y,et.al2010	12	90	23	70	17.1%	-0.20 [-0.33, -0.06]	2010
Wang,Y,et.al2012	7	36	15	36	7.8%	-0.22 [-0.43, -0.02]	2012
Xu,Y,et.al2012	14	33	24	33	7.2%	-0.30 [-0.53, -0.08]	2012
Zhang,Y2012	6	41	11	42	9.0%	-0.12 [-0.29, 0.06]	2012
Wang,J2012	4	25	7	24	5.3%	-0.13 [-0.36, 0.10]	2012
Xu,H,et.al2013	4	38	10	42	8.7%	-0.13 [-0.29, 0.03]	2013
Liu,Y,et.al2014	26	43	28	43	9.3%	-0.05 [-0.25, 0.16]	2014
**Total (95% CI)**		**476**		**448**	**100.0%**	**-0.13 [-0.18, -0.08]**	
Total events	165		215				

Heterogeneity: Chi² = 12.05, df = 10 (P = 0.28); I² = 17%

Test for overall effect: Z = 4.94 (P < 0.00001)

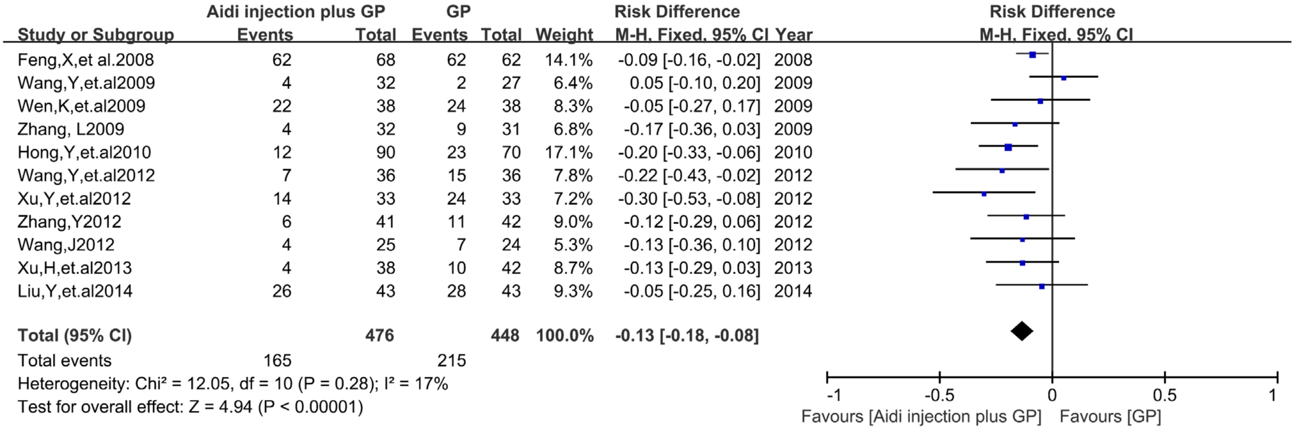

Supplementary Figure S3: The analysis of thrombocytopenia between two groups.

Study or Subgroup	Aidi injetion plus GP Events	Aidi injetion plus GP Total	GP Events	GP Total	Weight	Risk Difference M-H, Fixed, 95% CI
Hong,Y,et.al2010	8	90	12	70	27.9%	-0.08 [-0.19, 0.02]
Sun,J,et.al2012	6	34	7	34	12.1%	-0.03 [-0.22, 0.16]
Wang,J2012	5	25	5	24	8.7%	-0.01 [-0.23, 0.22]
Wang,Y,et.al2009	2	32	1	27	10.4%	0.03 [-0.08, 0.14]
Wang,Y,et.al2012	5	36	7	36	12.8%	-0.06 [-0.23, 0.12]
Wen,K,et.al2009	4	38	6	38	13.5%	-0.05 [-0.20, 0.10]
Zhang,Y2012	8	41	9	42	14.7%	-0.02 [-0.19, 0.15]
**Total (95% CI)**		**296**		**271**	**100.0%**	**-0.04 [-0.10, 0.02]**
Total events	38		47			

Heterogeneity: Chi² = 2.21, df = 6 (P = 0.90); I² = 0%
Test for overall effect: Z = 1.39 (P = 0.16)

Risk Difference
M-H, Fixed, 95% CI

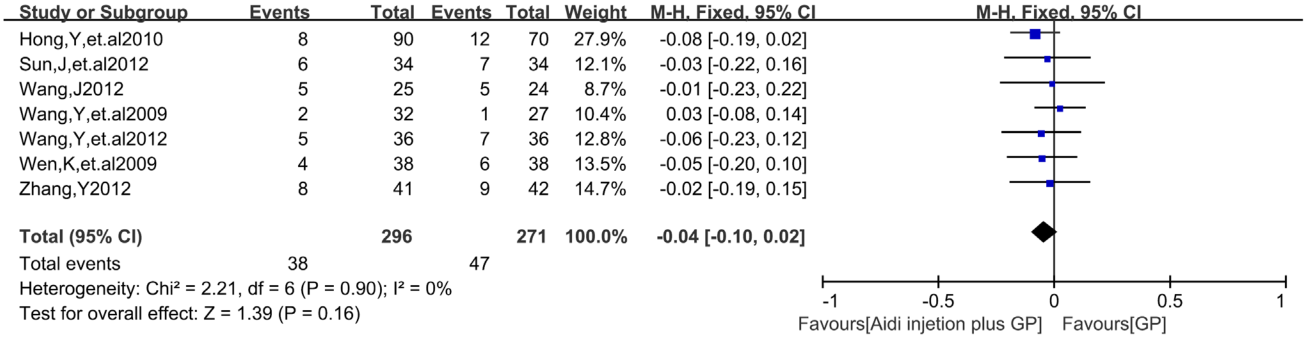

Supplementary Figure S4: The analysis of liver injury between two groups.

Study or Subgroup	Aidi injetion plus GP Events	Aidi injetion plus GP Total	GP Events	GP Total	Weight	Risk Difference M-H, Fixed, 95% CI
Hong,Y,et.al2010	4	90	7	70	36.3%	-0.06 [-0.14, 0.03]
Sun,J,et.al2012	3	34	4	34	15.7%	-0.03 [-0.17, 0.11]
Wang,J2012	4	25	4	24	11.3%	-0.01 [-0.21, 0.20]
Wen,K,et.al2009	2	38	4	38	17.5%	-0.05 [-0.17, 0.07]
Zhang,Y2012	5	41	6	42	19.1%	-0.02 [-0.17, 0.12]
**Total (95% CI)**		**228**		**208**	**100.0%**	**-0.04 [-0.10, 0.02]**
Total events	18		25			

Heterogeneity: Chi² = 0.38, df = 4 (P = 0.98); I² = 0%
Test for overall effect: Z = 1.35 (P = 0.18)

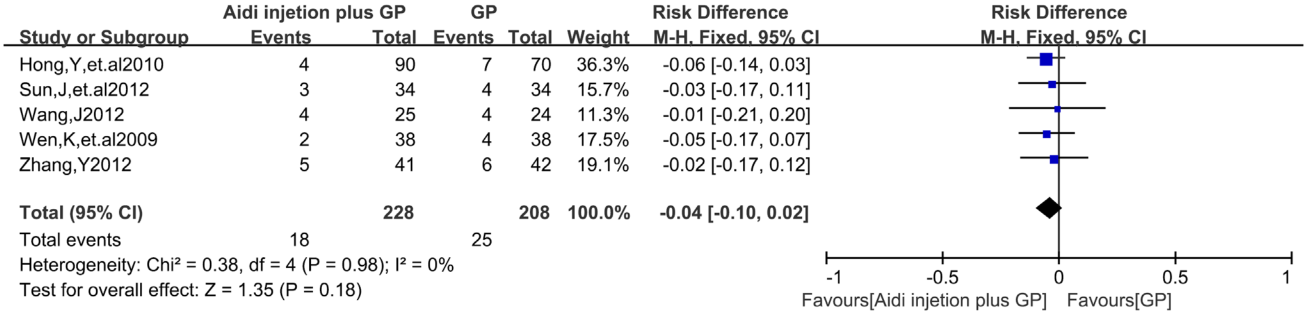

Supplementary Figure S5: The analysis of renal injury between two groups.

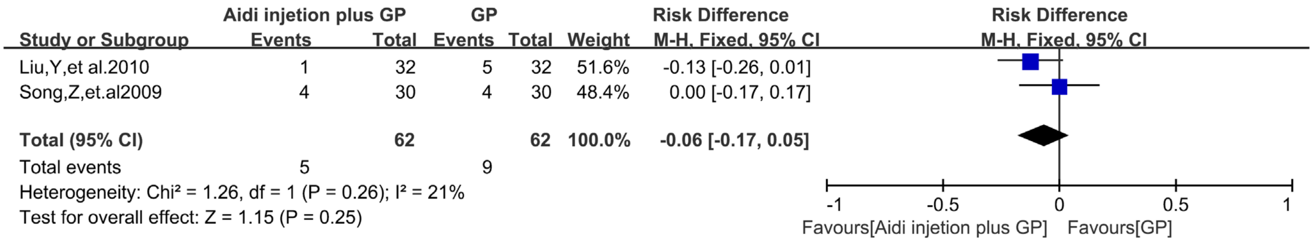

Supplementary Figure S6: The analysis of neurotoxicity between two groups.

	Aidi injetion plus GP		GP			Risk Difference	
Study or Subgroup	Events	Total	Events	Total	Weight	M-H, Fixed, 95% CI	Year
Feng,X,et al.2008	55	68	54	62	11.4%	-0.06 [-0.19, 0.06]	2008
Song,Z,et.al2009	10	30	11	30	5.3%	-0.03 [-0.27, 0.21]	2009
Lv,D,et.al2009	10	30	19	30	5.3%	-0.30 [-0.54, -0.06]	2009
Wen,K,et.al2009	20	38	29	38	6.7%	-0.24 [-0.45, -0.03]	2009
Wang,Y,et.al2009	5	32	5	27	5.2%	-0.03 [-0.22, 0.16]	2009
Shi,X,et.al2010	12	28	20	28	4.9%	-0.29 [-0.53, -0.04]	2010
Liu,Y,et al.2010	7	32	14	32	5.6%	-0.22 [-0.44, 0.00]	2010
Hong,Y,et.al2010	20	90	29	70	13.9%	-0.19 [-0.34, -0.05]	2010
He,W,et.al2011	12	29	13	23	4.5%	-0.15 [-0.42, 0.12]	2011
Wang,J2012	5	25	8	24	4.3%	-0.13 [-0.38, 0.11]	2012
Sun,J,et.al2012	28	34	29	34	6.0%	-0.03 [-0.20, 0.15]	2012
Zhang,Y2012	9	41	14	42	7.3%	-0.11 [-0.30, 0.08]	2012
Xu,Y,et.al2012	7	33	18	33	5.8%	-0.33 [-0.55, -0.11]	2012
Ju,S,et al2013	13	34	14	34	6.0%	-0.03 [-0.26, 0.20]	2013
Liu,Y,et.al2014	24	43	32	43	7.6%	-0.19 [-0.38, 0.01]	2014
**Total (95% CI)**		**587**		**550**	**100.0%**	**-0.15 [-0.21, -0.10]**	
Total events	237		309				

Heterogeneity: Chi² = 14.21, df = 14 (P = 0.43); I² = 1%
Test for overall effect: Z = 5.80 (P < 0.00001)

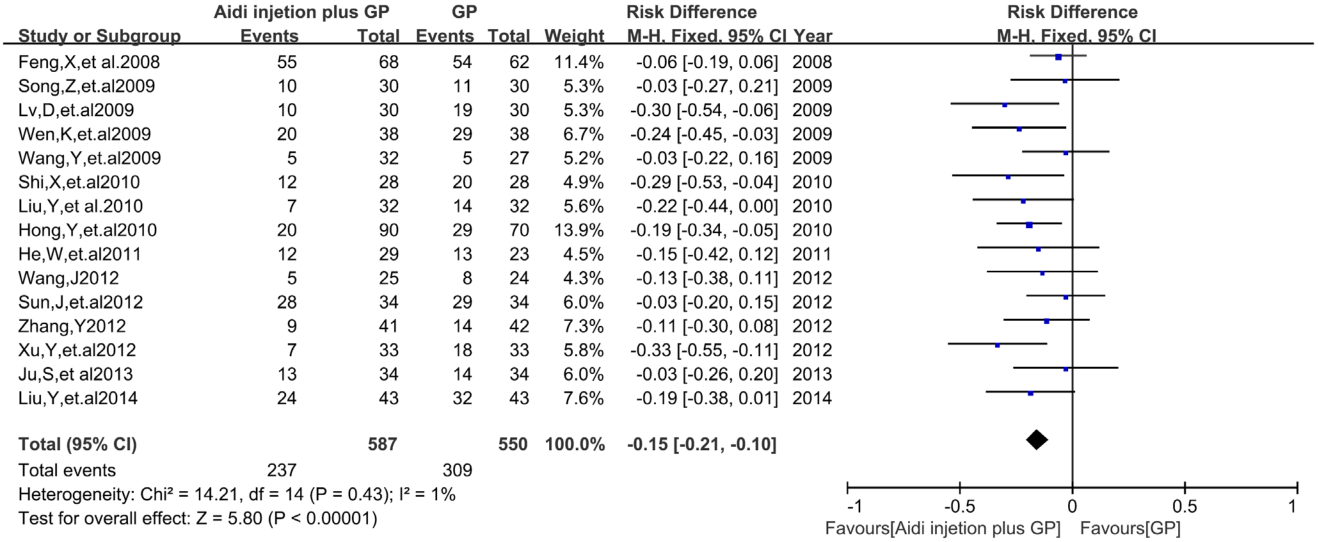

Supplementary Figure S7: The analysis of nausea and vomiting between two groups.
